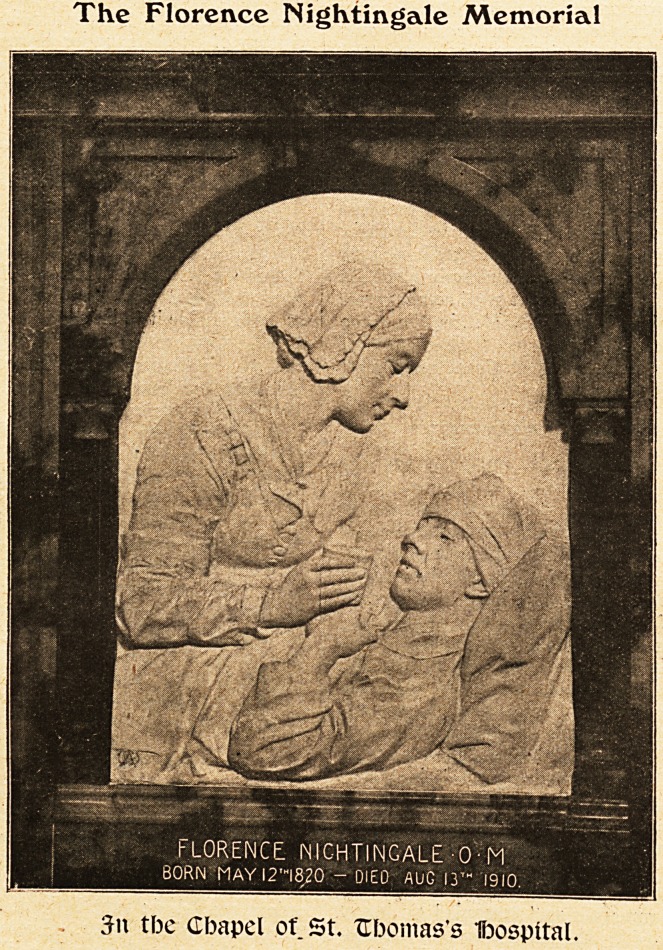# Unveiling of Bas-Relief of Florence Nightingale

**Published:** 1917-04-28

**Authors:** 


### UNVEILING OF BAS-RELIEF OF FLORENCE NIGHTINGALE.

A replica of the bas-relief erected as a memorial to Florence Nightingale in the crypt of St. Paul's has been executed by the sculptor, Mr. Arthur G. Walker, and erected in the chapel at St. Thomas's Hospital, Thames Embankment. On Wednesday, the 25th inst., this memorial was dedicated by His Grace the Archbishop of Canterbury. It was fitting that the chapel should be crowded with soldier patients and nurses, in addition to representative men and women closely associated with St. Thomas's Hospital and the Nightingale Fund and School. When we consider the marvels wrought daily in the hospitals, our thoughts travel back to the early years of St. Thomas's and to the sowing of the seed by Miss Nightingale of the art of nursing as it is now understood and practised.

In 1859 her intimate connection with St. Thomas's began. In that year came the opportunity for building the hospital on a healthier site and on an improved plan, and in carrying out this scheme none worked harder or to better purpose than Miss Nightingale. The South-Eastern Railway Company had found that the hospital, which was then in the Borough, would interfere with the projected extension of their line from London Bridge to Charing Cross. The resident medical officer, Mr. R. G. Whitfield, favoured total removal. He consulted Miss Nightingale, who not only agreed with him but also brought into action her entire battery of brain and influence. She placed the matter before the Prince Consort, who was one of the governors, and gained his whole-hearted support, and in spite of considerable opposition she eventually won the day. The hospital was removed temporarily to the Surrey Gardens, in 1868 Queen Victoria laid the foundation-stone of the new hospital, and in 1871 the well-known pavilioned building on the Albert Embankment was opened.

### The Florence Nightingale Memorial

*In the Chapel of St. Thomas's Hospital.*

The opening of the new hospital was a great event; but the opening of the Nightingale Training School for Nurses at St. Thomas's marked an epoch, for from this sprang the modern art and practice of nursing. Miss Nightingale had been greatly perplexed as to how to carry out her plans for applying the Nightingale Fund, which had been contributed by the nation in recognition of her services in the Crimea. Her original intention had been to found and conduct personally an institution for the training of nurses, but the state of her health and the demands of her Army work made this impossible. Finally, she came to the conclusion that the only satisfactory solution was to graft her institution upon some already existing establishment, and after much careful thought she decided upon St. Thomas's. The training-school was opened, and fifteen probationers were admitted on June 24, 1860, a day which is memorable in the annals of nursing. The

(*Continued on p. 74.*)

**FLORENCE NIGHTINGALE AND ST. THOMAS'S HOSPITAL**—(*Continued from p. 66.*)

fundamental ideas underlying the scheme were that the technical training should be the best possible, that the school should be a home as well as a school, and that probationers on completing the course should take up the career of hospital nursing, and thus spread the benefits of the training far and wide. Under the constant and careful supervision of its founder the institution flourished; Nightingale nurses carried their experience to the Overseas Dominions and the United States, to the Continent and elsewhere, and to-day every great hospital has its training-school for nurses.

Amidst her multifarious duties Miss Nightingale's interest in St. Thomas's never flagged; her heart was in the place. In 1864 she expressed the wish that when no longer able to work she should be taken to St. Thomas's and placed in a general ward; eight years later she had serious thoughts of entering the hospital as a patient, but was dissuaded by her friend Benjamin Jowett; and so intimate was her association with the hospital that in 1897, the news was widely but erroneously circulated, that, the invalid of South Street had been for many years an in-patient at St. Thomas's.

## Figures and Tables

**Figure f1:**